# Mr. Bartlett. on Monstrosity

**Published:** 1806-03

**Authors:** Michael Bartlett

**Affiliations:** Finsbury Dispensary, St. John's-Square


### *To the Editors of the Medical and Physical Journal.*

GENTLEMEN,

MY attention has been irresistibly led by a concatenation of plausible circumstances to the consideration of a subject which, in point of curious phenomena, affords one of the most interesting inquiries connected with the animal economy; I mean, that species of monstrosity called *Nævæ Maternæ*, or mother's marks, as they are vulgarly termed. It is a generally received opinion, that these appearances take place in consequence of impressions made on the mind of the mother, and conveyed, by mean of the nervous system, to the fœtus. That this opinion is founded in error, it is my object in the following observations to prove; in this attempt I know that I have not only to combat popular opinion, but likewise that of many reputable medical practitioners, with whom I have the honour to be acquainted.—The utter impossibility of such effects taking place from such a cause I do most steadily contend for, and that for the following reasons: There is no nervous communication wha ever, direct or indirect, between the mother and foetus; and as all sensation must be conveyed through the medium of the nerves, impressions made (however strong) upon the mother's mind, cannot affect the child. That there is no nervous communication is evident from the circumstance, that you may commit any act of violence upon the funis umbilicalis, without exciting sensation either in parent or child. That there is no other source of communication between the fœtal and maternal parts, is known to every person who has attended to the anatomy of the uterine system, as the placenta cannot be injected from the uterine vessels, or the uterus from the placenta, not even by the most subtle injection that we know of. That all the parts of an animal are completely formed *ab origine*, we have every reason to believe, though its extreme minuteness and fluidity conceal it for a time from our sight; it is an evolution of, and not a subsquent conformation of parts which takes place; for at a very early period we are enabled, by means of a microscope, to discover the rudiments of the fœtus. Now to apply this to my present purpose; if an animal body is completely formed in embryo, how is it possible that parts should be added or removed by impressions made upon the mother's mind at an advanced period of pregnancy. I will relate a case in point: A lady, distantly related to my family, had a son born with one arm only; the reason she assigned for this deformity was, that when she was about six months advanced in her pregnancy, a mendicant sailor, whose arm had been amputated near the shoulder, thrust his stump in her face, with a view to extort alms; this made such an impression upon her mind, that she attributed the dismemberment of her child to that cause. Allowing this to be possible, what is to become of the member so removed? It cannot escape out of the uterus, except per vaginam, and remaining there, it will act as an extraneous body, the consequence of which must soon be evident; what then is the cause? this is a question involved in too much obscurity for me to attempt to unravel; it is physiological, which perhaps, like the theory of conception, may never satisfactorily be explained; I can only say, that nature is not equally perfect in all her operations; we we see both in plants and animals considerable deviations from her usual standard. It is not peculiar to the human species; it takes place both in oviparous and viviparous animals, equally frequent in the brute as in man; neither is it the external part alone which becomes subject to these varieties, the internal organization is often found to be defective; there have been instances where only one kidney existed, where the gall bladder was wanting, where a child has been born at the full time of utero gestation and of full growth, without the least particle of osseous matter in its whole system.* This surely could not take place from imaginary causes; how it does take place I know not; but of this I am firmly persuaded, that let the appearance be what it may, that appearance took its rise at the very moment of conception.

Should these observations stimulate further inquiry in any of your more intelligent correspondents, I shall conceive myself amply rewarded for my trouble.

I am, &c.

*Finsbury Dispensary,*
*St. John's-Square, Feb.* 14, 1806.

MICHAEL BARTLETT.

* This preparation, which is I believe unique, is in the museum of my fiend Mr. Taunton.

